# Multimodal correlates of socioemotional movie-watching and their associations with internalizing symptoms in childhood and adulthood

**DOI:** 10.1093/scan/nsag050

**Published:** 2026-06-23

**Authors:** Sofia Scatolin, Elena Federici, Plamina Dimanova, Réka Borbás, Mirjam Habegger, Nora M Raschle

**Affiliations:** Jacobs Center for Productive Youth Development, Department of Psychology, University of Zurich, Zurich, 8050, Switzerland; Jacobs Center for Productive Youth Development, Department of Psychology, University of Zurich, Zurich, 8050, Switzerland; Neuroscience Center Zurich, University of Zurich and ETH Zurich, Zurich, 8057, Switzerland; Jacobs Center for Productive Youth Development, Department of Psychology, University of Zurich, Zurich, 8050, Switzerland; Jacobs Center for Productive Youth Development, Department of Psychology, University of Zurich, Zurich, 8050, Switzerland; Jacobs Center for Productive Youth Development, Department of Psychology, University of Zurich, Zurich, 8050, Switzerland; Neuroscience Center Zurich, University of Zurich and ETH Zurich, Zurich, 8057, Switzerland; Jacobs Center for Productive Youth Development, Department of Psychology, University of Zurich, Zurich, 8050, Switzerland; Neuroscience Center Zurich, University of Zurich and ETH Zurich, Zurich, 8057, Switzerland

**Keywords:** movie-watching, fMRI, heart rate, emotion, affect, development

## Abstract

Socioemotional skills emerge from coordinated behavioral, autonomic, and neural processes that continue to reorganize across development, yet how these systems jointly support emotion processing and mental health remains unclear. Using a naturalistic movie-watching paradigm, we integrated behavioral, cardiac, and fMRI measures in children (6–14 years) and adults (18–29 years), alongside independent ratings of experienced valence and arousal. Across age groups, positive and negative emotional content elicited changes in subjective experience, heart rate, and corticolimbic activity, including the amygdala, hippocampus, and prefrontal cortex. Despite these shared patterns, group differences emerged. Children reported more positive affect, showed larger heart-rate deceleration, and exhibited stronger recruitment of thalamic and lateral prefrontal regions, areas previously linked to sensory integration and cognitive control. Adults, in contrast, showed greater activation in hippocampal and posterior midline regions, which have previously been associated with memory and self-referential appraisal. During negative emotional content specifically, children preferentially engaged medial prefrontal regions, whereas adults engaged lateral prefrontal regions. Importantly, in adults but not children, models combining behavioral, cardiac, and neural indices explained substantially more variance in internalizing symptoms than any single modality alone. Together, these findings demonstrate that socioemotional experiences evoke coordinated behavioral, autonomic, and neural responses across development, while also revealing age-group differences in their organization and associations with mental well-being.

## Introduction

Socioemotional functioning emerges from the coordinated interaction of behavioral, autonomic, and neural systems and plays a central role in mental health and well-being across the lifespan. These systems support emotion processing and regulation in everyday life, yet how they jointly relate to vulnerability to psychopathology remains poorly understood. This coordination is particularly critical during development, when rapid changes in emotional experience, physiological regulation, and brain maturation may confer both resilience and risk for mental health difficulties.

Socioemotional skills encompass the ability to process and regulate emotions and are crucial determinants of key life outcomes and mental well-being. Research has linked these skills to distinct behavioral, cardiac, and neural correlates (James 1884, Lang *et al*. 1993, 1998, Damasio *et al*. 2000, Gross and John 2003, Barrett 2004, Chanes and Barrett 2016, Jauniaux *et al*. 2020, Moodie *et al*. 2020). However, much of this evidence has accumulated in a modality-specific manner, preventing the evaluation of changes within individual systems and their coordinated reorganization across systems. Alterations in socioemotional skills in different systems have been reported for various psychopathologies, including emotion processing and regulation difficulties in internalizing disorders (Browning *et al*. 2010, Berking *et al*. 2013, Chalmers *et al*. 2014). Notably, developmental studies integrating behavioral, cardiac, and neural measures remain rare, especially those incorporating neuroimaging (Raschle *et al*. 2012, Thieba *et al*. 2018, Barkovich *et al*. 2019).

Behaviorally, socioemotional skills are commonly assessed through subjective reports of emotional experience, typically characterized across dimensions of valence (unpleasant to pleasant) and arousal (deactivation to activation), while emotion regulation refers to the strategies individuals use to modulate these experiences. Emotion processing and regulation develop interactively and are tightly interdependent across childhood and adolescence (Petro *et al*. 2021). Early in development, emotional experiences are often represented in dichotomous terms, with greater emphasis on valence (Wintre and Vallance 1994, Larsen *et al*. 2007, Widen 2013). With increasing age, children report more nuanced emotional states integrating both valence and arousal dimensions (Larsen *et al*. 2007, Nook *et al*. 2017). Although subjective reports provide critical insight into emotional experience, they capture only part of the socioemotional process and cannot disentangle experiential changes from underlying physiological and neural mechanisms.

Emotion regulation skills follow a protracted developmental trajectory, paralleling broader cognitive and neurobiological maturation. While rudimentary regulatory behaviors appear early in life (e.g. gaze aversion; Ekas *et al*. 2013), the flexible and strategic use of cognitive emotion regulation strategies continues to develop from childhood through young adulthood (Tottenham *et al*. 2011, Gee and Cohodes 2021). These developmental changes coincide with advances in language, executive functioning, and prefrontal control, which have been associated with increasingly flexible modulation of emotional experience (Nook *et al*. 2017).

Emotion regulation strategies are commonly categorized as adaptive (e.g. acceptance, positive reappraisal, refocusing on planning) or maladaptive (e.g. rumination, catastrophizing, self-blaming). Although both influence emotional experience, they differ markedly in their implications for mental health. Adaptive strategies are generally associated with resilience and well-being, whereas maladaptive strategies predict poorer mental health outcomes, though contextual considerations apply (Aldao *et al*. 2010, Gratz *et al*. 2015, Dimanova *et al*. 2023). Overall, greater reliance on maladaptive regulation strategies has been consistently linked to elevated internalizing symptoms in both children and adults (Williams *et al*. 2007, Berking *et al*. 2008, Petro *et al*. 2021).

Physiologically, emotion processing and regulation are linked to variations in autonomic responses, including changes in heart rate reactivity and heart rate variability (HRV; fluctuation in time intervals between consecutive heartbeats; Brosschot and Thayer 2003, Pollatos *et al*. 2007, Nakahara *et al*. 2009, Wu *et al*. 2019). Across development, children typically show stronger heart rate reactivity to emotional stimuli than adults, while baseline HRV follows a protracted developmental trajectory reflecting ongoing maturation of autonomic control (Bar-Haim *et al*. 2000, Mather and Thayer 2018, Koenig 2020). Despite extensive research, the functional interpretation of cardiac responses during emotion processing remains debated, as autonomic responses are shaped by interacting aspects of emotional experience, including valence, arousal, and regulatory demands (Kreibig 2010). Importantly, lower HRV has been consistently linked to emotion regulation difficulties and elevated internalizing symptoms across development, highlighting autonomic functioning as a potential pathway linking emotion regulation and mental health (Koenig *et al*. 2016, Mulcahy *et al*. 2019, Heiss *et al*. 2021, Cainelli *et al*. 2022).

Neurally, socioemotional skills are linked to activity within a distributed corticolimbic network, encompassing prefrontal, temporal, and insular cortices as well as subcortical structures including the amygdala, hippocampus, and thalamus (Kohn *et al*. 2014, Morawetz *et al*. 2020). Within this network, subcortical regions tend to show greater engagement during emotional reactivity, whereas prefrontal regions are more strongly recruited during cognitive control and appraisal, though these patterns reflect the relative contributions of interacting systems rather than strict functional boundaries (Barrett and Satpute 2013, Chin *et al*. 2023). Across development, the functional integration of these systems strengthens, supported by increasing connectivity between prefrontal and subcortical regions, enabling progressively more flexible and effective regulation of affective responses (Gee *et al*. 2013, Morawetz *et al*. 2017, Raschle *et al*. 2019). Disruptions in the corticolimbic circuit are consistently implicated in internalizing psychopathology, highlighting altered neural coordination as a potential pathway underlying vulnerability to internalizing symptoms (Siegle *et al*. 2007, Beesdo *et al*. 2009, Wang *et al*. 2019).

Previous studies have frequently relied on task-based paradigms to assess socioemotional processes. Although these paradigms provide rigorous experimental control, they often fail to capture the dynamic nature of emotional experiences, may not fully account for effects occurring outside controlled conditions, and can reduce engagement, especially in children (Vanderwal *et al*. 2019, Cantlon 2020). Movie-watching paradigms help address these limitations by providing immersive and dynamic stimuli that require continuous integration of information (Hasson *et al*. 2009, Bottenhorn *et al*. 2019, Vanderwal *et al*. 2019). As such, they can evoke authentic emotions resembling real-world situations, eliciting behavioral, cardiac, and neural responses across development (Hasson *et al*. 2004, von Leupoldt *et al*. 2007, Codispoti *et al*. 2008, Bottenhorn *et al*. 2019, Sonkusare *et al*. 2019, Park *et al*. 2022). Additionally, movie-watching paradigms may improve neuroimaging data quality by reducing head motion (Vanderwal *et al*. 2019).

Despite evidence for developmental differences in behavioral, cardiac, and neural correlates of socioemotional processing, these modalities are typically studied in isolation. The present study therefore adopts a multimodal approach, jointly examining behavioral, cardiac, and neural responses during socioemotional movie watching in children and adults, and testing whether their integration explains additional variance in internalizing symptoms beyond any single modality. We hypothesize that: (i) emotion processing will manifest in systematic variations in self-reported valence and arousal, with children reporting more positive affect and lower arousal than adults (Nook *et al*. 2017, Buecker *et al*. 2023); (ii) cardiac responses will index heightened emotional reactivity, particularly in children (Kreibig 2010, Wrzus *et al*. 2014); (iii) neural responses will engage corticolimbic circuits previously implicated in emotion processing and regulation (Lindquist *et al*. 2012, Kohn *et al*. 2014), with children showing relatively greater subcortical and reduced prefrontal recruitment compared to adults (Rubia 2013, Larsen and Luna 2018); and (iv) integrating behavioral, cardiac, and neural measures will explain more variance in internalizing symptoms than any single modality, in both age groups.

## Materials and method

The study was pre-registered (https://osf.io/4kqha) and comprised two complementary components: Study Part 1 assessed subjective affect ratings during socioemotional processing using a movie-watching paradigm. Study Part 2 examined behavioral, cardiac, and neural responses using the same paradigm in an independent sample. Ethical approval was obtained from the Ethics Committee of the Canton of Zurich (BASEC-Nr. 2021-02096). Study Part 1 data were collected in schools or online (January–May 2024), whereas Study Part 2 participants completed an email/telephone-screening and an in-person session at the Children’s Hospital in Zurich (October 2022–October 2024).

### Participants

#### Study Part 1: behavioral affect ratings (valence and arousal)

A group of 69 children (44% female, age range 7–12 years, *M *= 9.88) and 218 adults (75.6% female, age range 18–34 years, *M *= 21.62) rated how they felt following the presentation of 17 scenes from the movie-watching paradigm (see *Movie-Watching Paradigm Evaluation* in the Supplementary Material). Procedural and consent details are provided in the Supplementary Material.

#### Study Part 2: behavioral, cardiac, and neural responses

Cardiac and neural responses during the movie-watching were collected from 109 children and 64 adults. All participants had normal or corrected-to-normal vision and sufficient German proficiency. Children showed average or above average IQ scores (>85; Table 1); IQ was not assessed in adults, all of whom were university students. Participants’ education and countries of origin are reported in the Supplementary Material.

**Table 1:** Demographic, behavioral, cardiac, and neural data of participants in Study Part 2.

		Children (*n* = 109, 47 female)				Adults (*n* = 64, 32 female)				Group difference *P*
		Mean	*SD*	Range	*n*	Mean	*SD*	Range	*n*	
Age	In years	10.01	2.31	6–14	109	22.8	2.3	18–29	64	
IQ	Total	112.4	10.8	87.5–142.5	109					
	Verbal	113.6	15.7	80–145	107					
	Nonverbal	110.9	12	85–140	109					
CERQ	Acceptance	2.91	1.02	1–5	75	3.51	1.03	2–5	64	<.001
	Positive refocus	2.97	1.16	1–5	75	2.04	0.83	1–5	64	<.001
	Refocus planning	3.03	1.14	1–5	75	3.66	1.03	1–5	64	<.001
	Positive reappraisal	2.51	0.94	1–5	75	3.59	1.15	1–5	64	<.001
	Put in perspective	2.75	0.95	1–5	75	2.92	1.19	1–5	64	.36
	Self-blame	2.37	0.80	1–4	75	2.51	0.87	1–4.5	64	<.001
	Rumination	2.38	1.04	1–5	75	3.06	0.84	1–5	64	<.001
	Catastrophizing	2.26	1.01	1–5	75	2.13	0.76	1–4.5	64	.39
	Other-blame	1.99	0.81	1–5	75	1.73	0.58	1–3.5	64	.03
SDQ	Total	7.1	4.8	0–22	104					
	Internalizing	2.5	2.7	0–13	104					
BSI	Total					49.1	9.2	26–68	64	
	Internalizing					51.1	7.3	38.3–68	64	
HRV	RRMSD	78.52	39.69	19–221.5	105	66.11	37.3	17.5–205.6	64	.04
Left vlPFC	In neg > pos	0.1981	0.29	–0.91 to 1.03	95	0.1884	0.21	–0.43 to 0.6	58	.76
Sup. PFC/SMA	In neg > pos	0.1812	0.34	–1.02 to 1.46	95	0.1523	0.18	–0.21 to 0.55	58	.50

*Note.* CERQ = short version of the Cognitive Emotion Regulation Questionnaire; SDQ = Strengths and Difficulties Questionnaire; BSI = Brief Symptom Inventory; HRV = heart rate variability; RRMSD = relative root-mean-squared differences; Sup. PFC/SMA = mean parameter estimates in the bilateral superior prefrontal cortex and supplementary motor area cluster; vlPFC = mean parameter estimates in the ventrolateral prefrontal cortex cluster; “In neg > pos” = during the “negative > positive emotion processing” contrast.

### Behavioral assessments

#### Study Part 1

Data were collected anonymously, with only sex and age recorded.

#### Study Part 2

Children completed verbal (vocabulary) and non-verbal (matrices) IQ assessments using the Wechsler Intelligence Scales for Children (Petermann and Petermann 2008). Parents reported on children’s mental well-being using the Strengths and Difficulties Questionnaire (SDQ; Goodman 1997). Internalizing symptoms scores were derived from the sum of the SDQ *Peer Problems* and *Emotional Problems* subscales.

Adults’ mental well-being was assessed through the Brief Symptoms Inventory (BSI; Derogatis 1993), with internalizing symptoms computed as the mean of the *Interpersonal Sensitivity*, *Depression*, and *Anxiety* subscales (*t*-scores adjusted for sex and age).

For adults and children aged 9 years and older, cognitive emotion regulation strategies were assessed through the short version of the Cognitive Emotion Regulation Questionnaire (CERQ-short; Garnefski and Kraaij 2006, Garnefski *et al*. 2007). The questionnaire includes five adaptive strategies (positive refocusing, acceptance, refocus on planning, positive reappraisal, and putting into perspective) and four maladaptive strategies (self-blame, rumination, catastrophizing, and other-blame) subscales. All child assessments were administered by trained research staff.

### Movie-watching paradigm

#### Design

A movie-watching paradigm was developed to assess socioemotional processing in children and adults. The paradigm consisted of scenes and promotional material from the Pixar Animation Studio’s movie *Inside Out* (Docter and Del Carmen 2015, Pixar 2015a, 2015b), complied into a coherent 12-min 29-s narrative containing a broad range of socioemotional content.

#### Evaluation

The content of the movie-watching paradigm was first analyzed using EmoCodes, an externally validated, frame-by-frame system designed to code the affective content of complex stimuli, via the emocodes Python library (Camacho *et al*. 2022). Three independent adult raters coded the emotions displayed by eight characters, encompassing seven categories: anger, sadness, fear, happiness, surprise, disgust, and shame. Inter-rater agreement was high (98.46%).

**Figure 1:**
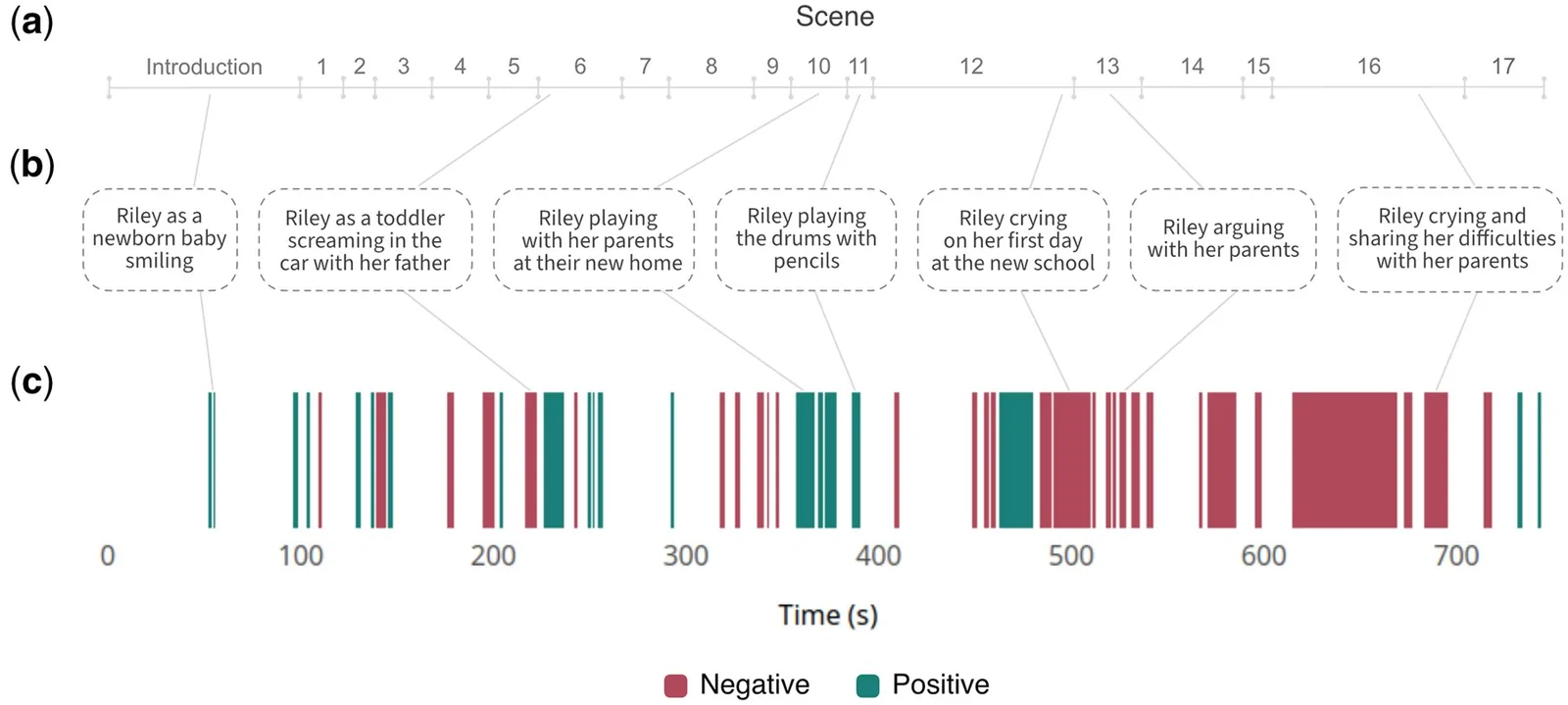
Movie-watching paradigm design. *Note*. (a) Seventeen discrete scenes from the movie-watching paradigm used in data collection of Study Part 1 (behavioral affect ratings). (b) Descriptions of selected narrative moments of the movie-watching paradigm. (c) Events in which the main character displayed positive and negative emotions, defined by raters using the EmoCodes system (Camacho *et al*. 2022).

For the present study, we identified events in which the main character displayed positive emotions (happiness and surprise) and negative emotions (anger, sadness, fear, disgust, and shame), based on unanimous rater agreement, and extracted their onsets and durations. This procedure yielded two regressors, “positive emotions” and “negative emotions,” which served as predictors in all analyses (Fig. 1; Supplementary Material).

For Study Part 1, the movie-watching paradigm was divided into 17 scenes ranging from 16 to 105 seconds (*M *= 37.82, *SD* = 27.46), based on the narrative structure so that each scene represented a thematically coherent, self-contained segment. After watching each scene, children and adults rated their experienced valence (from 1 to 5, 1=“unpleasant” and 5=“pleasant”), arousal (ranging from 1 to 5, 1=“deactivation” and 5=“activation”), and the specific emotions they personally experienced and perceived in the characters (happiness, surprise, fear, disgust, anger, sadness, shame, neutral; multiple selections permitted). Ratings were collected using child-friendly visual self-rating scales, including an adaptation of the Self-Assessment Manikin (Bradley and Lang 1994) modified to enhance visual appeal for younger participants, as well as emoji-based pictorial aids (see Supplementary Material).

### Neuroimaging and cardiac data acquisition

In Study Part 2, participants underwent fMRI and simultaneous photoplethysmography (PPG) while viewing the movie-watching paradigm. Detailed acquisition parameters are provided in the Supplementary Material.

### Analyses

#### Evaluation of behavioral affect: valence and arousal ratings (Study Part 1)

Linear mixed-effects models were used to assess the effects of positive and negative emotion events on valence and arousal ratings, with separate models for children and adults. Analyses were conducted in R (R Core Team 2025) using lme4 (Bates *et al*. 2023) and lmerTest (Kuznetsova *et al*. 2017).

For each of the 17 movie scenes, we calculated the duration (in seconds) of positive and negative emotional content, as assessed with EmoCodes (Camacho *et al*. 2022; see *Movie-Watching Paradigm Evaluation* in the Supplementary Material). Negative emotion durations ranged from 0 to 70 seconds (*M *= 20.39) and positive emotion durations ranged from 0 to 17 seconds (*M *= 5.14). Each model included a fixed intercept and fixed effects for positive emotion duration, negative emotion duration, and other content duration (i.e. segments without emotional displays by the main character), as well as scene mid-time (calculated as the midpoint of each scene, to account for time), age, and sex. Random intercepts for participants and random slopes for positive and negative emotion durations were included to model within-subject variability. All continuous predictors were z-standardized.

To assess group differences, participant-specific random intercept and slopes were extracted. Group differences (children vs. adults) were tested using t-tests.

#### Cardiac and neural correlates (Study Part 2)

##### Cardiac analyses

Cardiac data were missing for two children who declined to wear the pulse oximeter. PPG data were preprocessed in Python using NeuroKit2 (version 0.2.13, Makowski *et al*. 2021) and signal quality was assessed using the default method, which computes a continuous quality index (0–1) based on the correlation between each individual pulse wave and an average template waveform. Two child participants with mean quality indices ≤0.65 and ≥20% low-quality samples were excluded, yielding a final sample of 105 children and 64 adults for cardiac analysis. Signal quality was high in both groups (adults: *M *= 0.98, *SD* = 0.02; children: *M *= 0.97, *SD* = 0.04). Heart rate (beats per minute) was computed at 1 Hz. Linear mixed-effects models were run separately for children and adults to test the effects of positive and negative emotional events on heart rate. The model structure mirrored that used for the behavioral affect ratings (Study Part 1), except that positive and negative emotion events were coded as binary variables (present/absent) for each second, rather than as durations, because heart rate was sampled at 1 Hz rather than at the scene level. Time was therefore modeled continuously in seconds.

For the post-hoc analysis, HRV was quantified RMSSD (root mean square of successive differences), a standard HRV index (Pham *et al*. 2021), and was extracted using NeuroKit2 (Makowski *et al*. 2021) across the full duration of the movie-watching paradigm.

##### Neuroimaging analyses

Neuroimaging data were analyzed using SPM12 (The Wellcome Centre for Human Neuroimaging 2020) in MATLAB (MathWorks 2023). Preprocessing followed standard protocols: realignment and unwarping, co-registration with each participant’s structural image, and segmentation, normalization (ICBM152) and smoothing (6-mm full-width at half-maximum isotropic Gaussian kernel). Following visual inspection of all individual masks and image series, 14 children and 6 adults were excluded due to excessive head motion or magnetic artifacts from dental braces, resulting in a final sample of 95 children and 58 adults. Motion-related and global signal fluctuations were modeled using the Artifact Detection Tools (ART) toolbox (Whitfield-Gabrieli *et al*. 2011), which generated seven nuisance regressors for each first-level model. A high-pass temporal filter (cutoff = 0.01 Hz; 128 seconds) was applied.

Whole-brain analyses were conducted for the child and adult groups. First-level models included regressors for positive emotion, negative emotion, and other events (i.e. without emotional displays by the main character), as defined by the EmoCodes analysis (see *Design* and Supplementary Material).

Second-level analyses included one-sample t-tests for each group for positive emotion, negative emotion, negative > positive emotion, and positive > negative emotion events contrast, with age and sex as covariates. Statistical significance was assessed using cluster-level family-wise error (FWE) correction at *P* < .05, with an initial voxel-wise threshold of *P* < .001 (uncorrected). Clusters were labeled using the Automated Anatomical Atlas 3 (AAL3; Rolls *et al*. 2020). Conjunction analyses were performed with SPM’s ImCalc utility to identify shared activation across groups. Group differences were tested using two-sample t-tests.

#### Post hoc: associations between behavioral, cardiac, and neural correlates of emotion processing and internalizing symptoms (Study Part 2)

To test whether behavioral, cardiac, and neural measures were associated with internalizing symptoms and whether combining models across modalities improved explanatory power, multiple linear regression models were computed separately for 60 children and 58 adults with complete behavioral (i.e. cognitive emotion regulation strategies, CERQ), cardiac, and neuroimaging data, using olsrr (Hebbali 2024) in R. Five models were estimated per group, with internalizing symptoms as the outcome (SDQ for children; BSI for adults) and age and sex as covariates. All predictors and outcomes were scaled (centered and Z-standardized) before analyses. Because internalizing symptom scores in children were right-skewed, they were square-root-transformed prior to scaling and analyses.

The null model included only sex and age as predictors. The behavioral model tested whether cognitive emotion regulation strategies were associated with internalizing symptoms. Predictors were the CERQ adaptive (acceptance, positive refocusing, refocus on planning, positive reappraisal, putting into perspective) and maladaptive (self-blame, rumination, catastrophizing, other-blame) subscales. Stepwise forward selection was used to identify predictors that increased explained variance and began with sex and age. Additional predictors were entered sequentially based on the largest increase in adjusted *R*^2^, stopping when no further improvement occurred.

The cardiac model assessed whether HRV (RMSSD), extracted during the full movie-watching paradigm, was associated with internalizing symptoms.

The neural model assessed whether mean parameter estimates from the left vlPFC and superior PFC/SMA clusters identified in the negative > positive emotion processing conjunction analysis explained variance in internalizing symptoms. These regions were selected based on prior literature implicating lateral and superior prefrontal regions in emotion processing and regulation (e.g. Ochsner *et al*. 2012, Kohn *et al*. 2014, Raschle *et al*. 2025), and evidence that negative emotional stimuli are associated with greater prefrontal recruitment compared to positive stimuli (Morawetz *et al*. 2016, Alves *et al*. 2017). As in the behavioral model, stepwise forward selection began with age and sex, and predictors were added based on the largest increase in adjusted *R*^2^, stopping when no further improvements were obtained. If no predictor improved adjusted *R*^2^ during stepwise selection, supplementary models including each predictor separately were estimated, and the model with the highest adjusted *R*^2^ was retained for comparison purposes.

The multimodal model examined whether models combining behavioral, cardiac, and neural predictors explained additional variance in internalizing symptoms relative to single-modality models. Predictors included all CERQ subscales, HRV, and neural activity in left vlPFC and superior PFC/SMA. Stepwise forward selection again began with sex and age and added predictors based on the largest adjusted *R*^2^ gain, continuing until there were no more gains. Model performance was evaluated relative to the single-modality (i.e. behavioral, cardiac, and neural) models using AIC, adjusted *R*^2^, and ANOVA. Because these analyses were exploratory, stepwise selection was used to reduce model dimensionality. Models including all predictors were additionally estimated to assess robustness (see Supplementary Material). To characterize associations among multimodal predictors, Pearson correlations were computed between all behavioral, cardiac, and neural variables, separately for children and adults.

##### Deviations from preregistration

First, directional hypotheses were refined based on the literature after pre-registration, and should therefore not be interpreted as pre-registered hypotheses. Second, analyses examining associations between behavioral, cardiac, and neural measures and internalizing symptoms were added post-hoc and are reported as exploratory. Third, some preregistered analyses were not conducted because they exceeded the scope of the present article.

## Results

### Evaluation of affect: valence and arousal ratings (Study Part 1)

**Figure 2:**
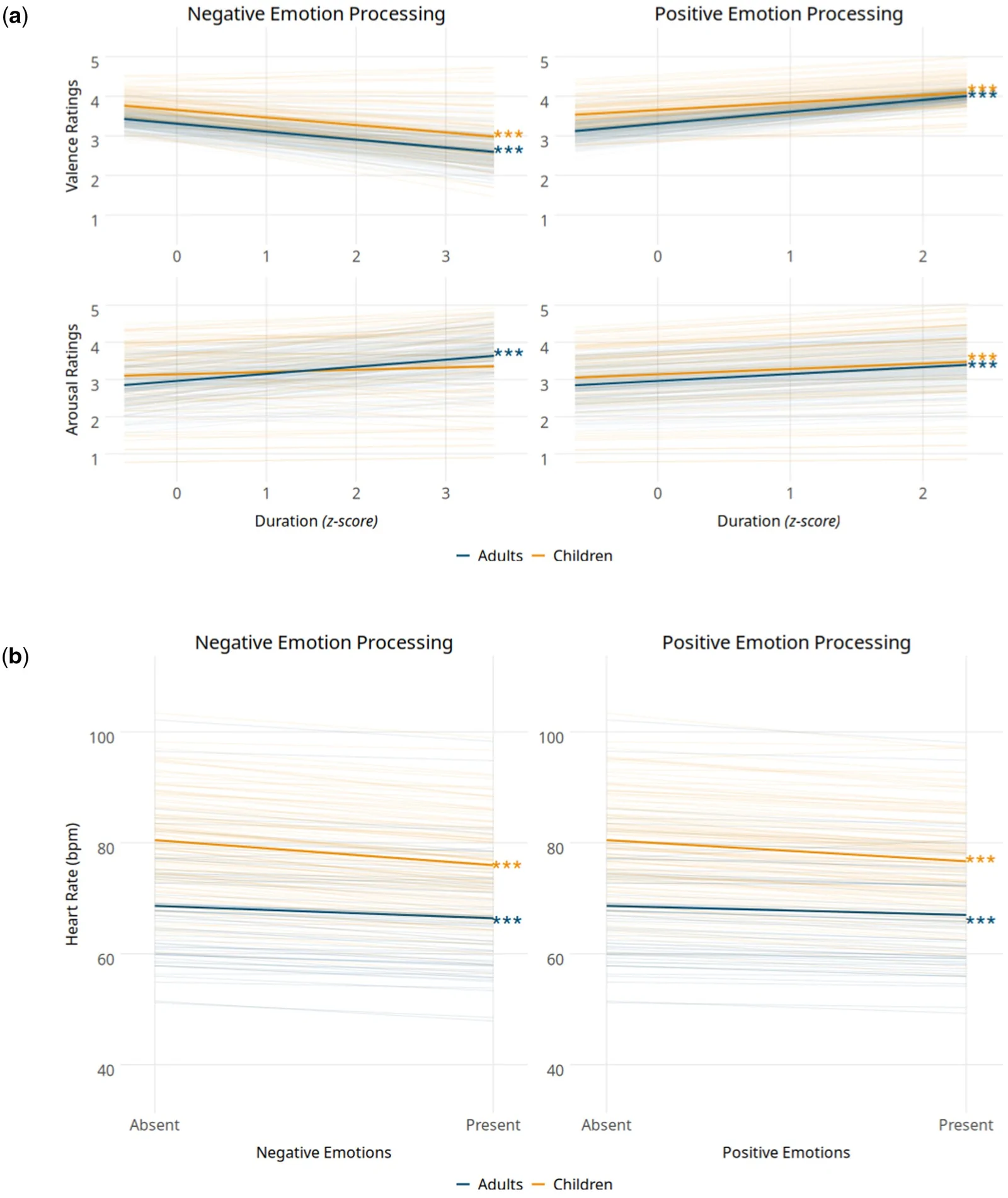
Behavioral and cardiac correlates of emotion processing. *Note*. (a) Changes in participants’ valence and arousal ratings paralleling the duration of negative and positive emotion events displayed in each movie scene. Valence ratings range from 1 to 5, with 1 corresponding to unpleasant and 5 to pleasant. Arousal ratings range from 1 to 5, with 1 corresponding to deactivation and 5 to activation. (b) Changes in heart rate paralleling the presence or absence of positive and negative emotion events at each second. Positive and negative emotion events, as well as other content, were included as separate predictors in the same model, so the plotted effects reflect the association of each emotion with heart rate while adjusting for the others. In each plot, “absence” refers to seconds without that specific emotion. (a and b) Thinner lines represent individual data and bolder lines indicate group effects. ***P < .001.

Positive and negative emotional content was associated with variations in participants’ valence ratings in both age groups. In children, longer durations of negative emotion events decreased valence (*B *=* −*0.19, *SE *= 0.04, *P* < .001, 95% CI [*−*0.27, *−*0.10]), while longer durations of positive emotion events increased valence (*B *= 0.19, *SE *= 0.03, *P* < .001, 95% CI [0.13, 0.25]), such that longer durations of negative emotional content were associated with lower valence ratings, whereas longer durations of positive emotional content were associated with higher valence ratings. In adults, a similar pattern was observed: longer durations of negative emotion events decreased valence (*B *=* −*0.20, *SE *= 0.02, *P* < .001, 95% CI [*−*0.25, *−*0.15]) and positive emotion durations increase valence (*B *= 0.30, *SE *= 0.02, *P* < .001, 95% CI [0.26, 0.34]). Between-group comparisons indicated that children exhibited higher valence at average values of negative and positive emotion durations (i.e. intercept) than adults (*t*(83.19) = 6.94, *P* < .001), whereas adults exhibited steeper increases in valence with longer durations of positive emotion events (i.e. slopes) than children (*t*(184.9)=−23.17, *P* < .001; Fig. 2a).

Emotion events were also associated with variations in participants’ arousal ratings. In children, positive, but not negative, event durations were associated with increased arousal (*B*_negative_ = 0.06, *SE*_negative_ = 0.04, *P* = .15, 95% CI [*−*0.02, 0.14]; *B*_positive_ = 0.14, *SE*_positive_ = 0.03, *P* < .001, 95% CI [0.08, 0.20]). In adults, both negative and positive emotion event durations increased arousal (*B*_negative_ = 0.19, *SE*_negative_ = 0.02, *P* < .001, 95% CI [0.15, 0.23]; *B*_positive_ = 0.18, *SE*_positive_ = 0.01, *P* < .001, 95% CI [0.16, 0.22]). Adults showed steeper increases in arousal with longer durations of both positive and negative emotion events (i.e. slopes) than children (*t*(105.46)_negative_ = *−*7.65, *t*(109.24)_positive_ = *−*5.83, both *P* < .001; Fig. 2a).

### Behavioral, cardiac, and neural correlates of emotion processing (Study Part 2)

#### Group characteristics

Descriptive statistics and group comparisons are presented in Table 1.

#### Cardiac correlate

During movie watching, both positive and negative emotion events were associated with heart rate decreases in children (*B*_negative_ = −4.51, SE_negative_ = 0.54, *P* < .001, 95% CI [−5.56, −3.45]; *B*_positive_ = −3.81, SE_positive_ = 0.54, *P* < .001, 95% CI [−4.87, −2.76]) and adults (*B*_negative_ = −2.23, SE_negative_ = 0.47, *P* < .001, 95% CI [−3.15, −1.31]; *B*_positive_ = −1.64, SE_positive_ = 0.46, *P* < .001, 95% CI [−2.55, −0.73]; Fig. 2b). Between-group comparisons indicated that children exhibited higher heart rate in the absence of negative and positive emotion events (i.e. intercept) than adults (*t*(129.46) = 8.44, *P* < .001). Children showed significantly larger heart rate decreases (i.e. slopes) than adults during both negative (*t*(147.66) = −11.385, *P* < .001) and positive (*t*(161.55 = −11.659, *P* < .001) emotion events.

#### Neural correlates

**Figure 3:**
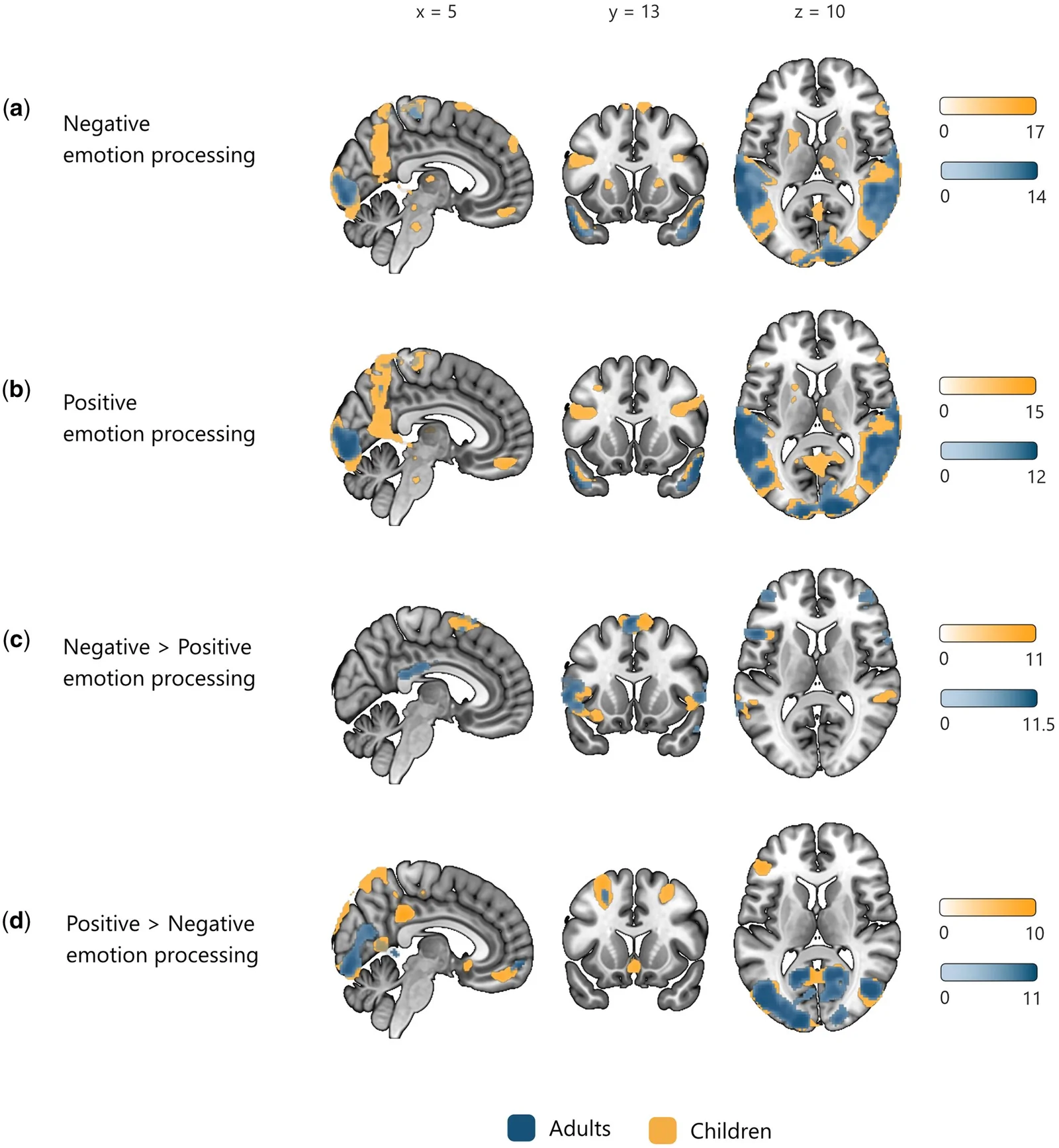
Neural correlates of emotion processing. *Note*. Statistical parametric maps showing neural activation during negative emotion events (a), positive emotion events (b), negative > positive emotion events (c), and positive > negative emotion events in children and adults (d). Maps are cluster-level FWE-corrected *P* < .05, using a cluster-building threshold of *P* < .001 (uncorrected).

In both children and adults, one-sample *t*-tests revealed that positive and negative emotional content was associated with increased activation across frontal, temporal, parietal, occipital, and limbic regions, including the prefrontal cortex (PFC), temporoparietal junction (TPJ), amygdala, and hippocampus (Fig. 3a and b; Tables 2 and 3). Conjunction analyses confirmed overlapping activation patterns across groups. The contrast negative > positive emotion events was associated with greater activation in dorsolateral frontal, superior frontal, and temporal regions in both groups (Fig. 3c; Tables 2 and 3). Conjunction analyses further revealed shared clusters in the left ventrolateral PFC (vlPFC), superior PFC/supplementary motor area (SMA), and bilateral temporal regions. The contrast positive>negative emotion events was associated with greater activation in ventromedial frontal, superior frontal, occipital, parietal, temporal, and the hippocampus in both groups (Fig. 3d; Tables 2 and 3), which was confirmed in conjunction analyses. The precise regions identified in the conjunction analyses, according to AAL3, are listed in the Supplementary Material.

**Table 2:** Peak activations for neural activation during negative, positive, negative > positive, and positive > negative emotion processing in the child group.

Brain region	Hem.	*T*	*p*fwe	*k*	MNI		
					*x*	*y*	*z*
**Negative emotion processing**							
Inf., mid., and sup. temporal gyri; mid. and sup. temporal pole; triangular, orbital, and operational inferior frontal gyri; lateral and posterior OFC; inf., mid., and sup. occipital gyri; fusiform, calcarine, lingual, supramarginal, angular, parahippocampal, postcentral gyri; thalamus, hippocampus, amygdala; cuneus; cerebellum and vermis	L/R	16.96	0	29 203	–60	–18	–4
Cuneus, precuneus, paracentral lobule, supplementary motor area; calcarine, lingual, precentral, and postcentral gyri; mid. and post. cingulate gyri; sup. parietal gyrus; and vermis	L/R	8.78	0	2284	–4	–56	36
Medial orbital frontal gyrus; medial OFC; and rectus	L/R	7.79	0.004	245	4	50	–20
Triangular, orbital, and operational inferior frontal gyri; lateral and posterior OFC; sup. temporal pole; and precentral gyrus	L	6.91	0	824	–54	24	–2
Medial and dorsolateral sup. frontal gyri; supplementary motor area	L/R	6.48	0	659	–8	56	42
Precentral, postcentral, supramarginal, triangular and operational inf. frontal; mid. and dorsolateral sup. frontal gyri	R	6.45	0	736	52	6	52
Putamen; pallidum; and caudate	L/R	6.42	0	406	–22	–2	4
Supplementary motor area; medial and dorsolateral sup. frontal gyri	R	6.25	0.004	237	8	16	70
Thalamus; hippocampus; parahippocampal; and lingual gyri	L	6.07	0.007	215	–16	–32	–4
Precentral and mid. frontal gyri	L	6.03	0.005	234	–46	–0	56
Putamen, pallidum, caudate	R	5.34	0.002	282	24	–4	2
							
**Positive emotion processing**							
Inf., mid., and sup. temporal gyri; mid. and sup. temporal pole; inf., mid., and sup. occipital; inf. and sup. parietal gyri; anterior and posterior OFC; mid. and post. cingulate gyri; fusiform, calcarine, lingual, supramarginal, angular, precentral, postcentral, and parahippocampal gyri; thalamus, hippocampus, amygdala; paracentral lobule, supplementary motor area; precuneus; cuneus; cerebellum; and vermis	L/R	14.39	0	35 185	64	–10	–2
Medial orbital frontal gyrus; medial OFC; and rectus	L/R	8.92	0	445	4	50	–20
Triangular and orbital inferior frontal gyri	R	7.03	0.013	197	58	26	2
Precentral gyrus; triangular and operational inf. frontal gyri; mid. and dorsolateral sup. frontal gyri; supplementary motor area	R	6.55	0	1730	40	18	26
Thalamus, hippocampus, and parahippocampal gyri	L	6.43	0.009	214	–16	–32	–4
Triangular, orbital, and operational inf. frontal gyri; posterior and lateral OFC; precentral gyrus; and sup. temporal pole	L	6.11	0	788	–48	16	24
Medial and dorsolateral sup. frontal gyri	L	5.79	0	367	–12	50	46
Precentral gyrus; mid. and dorsolateral sup. frontal gyri; paracentral lobule; and supplementary motor area	L	5.58	0	1038	–44	–2	58
Thalamus; putamen; pallidum; and caudate	L	4.84	0.04	151	–22	–2	4
							
**Negative > positive emotion processing**							
Inf., mid., and sup. temporal gyri; inf. parietal, supramarginal and angular gyri; mid. and sup. temporal pole	L	10.95	0	3145	–48	–30	–4
Mid. and sup. temporal gyri; mid. and sup. temporal pole	R	9.85	0	1885	48	–24	–4
Triangular, orbital, and operational inf. frontal gyri; insula; and posterior OFC	L	7.11	0	971	–48	20	–6
Mid., and sup. temporal, inf. parietal, supramarginal, and angular gyri	R	6.67	0	519	60	–52	44
Supplementary motor area; medial and dorsolateral sup. frontal gyri	L/R	6.35	0	768	8	14	66
Insula; orbital, and operational inf. frontal gyri; sup. temporal pole	R	6.01	0.014	204	50	18	–6
Precuneus and cuneus	R	5.43	0.025	179	14	–62	34
							
**Positive > negative emotion processing**							
Mid. and sup. occipital gyri; inf. and sup. parietal gyri; hippocampus; precuneus; lingual, fusiform, calcarine, angular, supramarginal, postcentral, and parahippocampal gyri; mid. cingulate; cerebellum and vermis	L/R	9.87	0	24 388	–12	–22	40
Mid., sup., and operational inf. frontal gyri; precentral gyrus; supplementary motor area	L	8.66	0	2868	–24	–12	52
Medial orbital frontal gyrus; medial OFC; rectus	L/R	6.61	0	497	–6	42	–16
Mid. and triangular inf. frontal gyrus	L	6.32	0	394	–44	30	18
Inf. and mid. temporal gyri	R	6.26	0.002	299	56	–44	–14
Olfactory gyri; ventral striatum; caudate	L/R	5.68	0.036	163	4	14	–10
Mid. and sup. frontal gyri; precentral gyrus; supplementary motor area	R	5.25	0	1253	26	–2	50

*Note.* Hem = hemisphere; OFC = orbitofrontal cortex; inf. = inferior; mid. = middle; sup. = superior; L/R = left/right; *T* = *T*-scores; *k* = cluster size; and *x*, *y*, *z* co-ordinates of peak voxel according to Montreal Neurological Institute (MNI).

**Table 3:** Peak activations for neural activation during negative, positive, negative > positive, and positive > negative emotion processing in the adult group.

Brain region	Hem.	*T*	*p*fwe	*k*	MNI		
					*x*	*y*	*z*
**Negative emotion processing**							
Inf., mid., and sup. temporal gyri; mid. and sup. temporal pole; inf., mid., and sup. occipital; triangular and orbital inf. frontal gyri; inf. parietal gyrus; lateral and posterior OFC; fusiform, supramarginal, angular, precentral, postcentral, and parahippocampal gyri; amygdala; hippocampus; cuneus; cerebellum	L/R	13.39	0	20 332	56	–26	–2
Amygdala; hippocampus; parahippocampal and fusiform gyri	L	6.61	0.009	141	–26	–6	–28
Thalamus; hippocampus; and lingual gyrus	R	6.46	0.001	210	16	–32	–2
Triangular and orbital inf. frontal gyri	R	5.88	0.001	193	58	32	4
Precentral; postcentral; and dorsolateral sup. frontal gyri	L	5.48	0.005	157	–36	–6	66
Paracentral lobule; precuneus; supplementary motor area; sup. parietal, postcentral, and precentral gyri	R	5.09	0	346	10	–34	72
Paracentral lobule; precuneus; and postcentral gyrus	L	5.00	0.001	216	–12	–36	80
							
**Positive emotion processing**							
Inf., mid., and sup. temporal gyri; mid. and sup. temporal pole; inf., mid., and sup. occipital gyri; fusiform, lingual, calcarine, supramarginal, angular, precentral, postcentral, and parahippocampal gyri; amygdala; cuneus; cerebellum; and vermis	L/R	11.86	0	21 675	60	–4	–10
Precentral, mid. and sup. frontal gyri	L	6.39	0	436	–36	–6	66
Hippocampus; amygdala; parahippocampal and fusiform gyri; mid. and sup. temporal pole	R	6.11	0	301	26	–2	–26
Thalamus; hippocampus and lingual gyrus	R	6.03	0.010	135	16	–32	–2
Precuneus and paracentral lobule; inf. and sup. parietal, precentral, and postcentral gyri	L/R	5.53	0	136	16	–60	–62
Triangular and orbital inf. frontal gyri	R	5.32	0.009	139	58	30	4
Dorsolateral and medial sup. frontal gyri	L	4.69	0.006	150	–16	40	54
							
**Negative > positive emotion processing**							
Inf., mid., and sup. temporal gyri; mid. and sup. temporal pole; insula	R	11.22	0	1807	56	–28	–2
Inf., mid., and sup. temporal gyri; sup. temporal pole	L	8.77	0	1641	–54	–36	–4
Triangular and orbital inferior, mid., and dorsolateral sup. frontal gyri	R	6.80	0	588	46	42	2
Inf. parietal, supramarginal, angular, and mid. and sup. temporal gyri	L	6.62	0	419	–60	–54	38
Triangular, orbital, and operational inferior, mid., and sup. frontal gyri; sup. temporal pole; lateral and posterior OFC; insula	L	6.59	0	1249	–54	16	2
Supplementary motor area; medial and lateral superior frontal gyri	L/R	6.24	0	487	–2	12	66
Mid. and post. cingulate gyri	L/R	5.51	0	383	4	–36	20
Inf. occipital, fusiform, lingual, and inf. temporal gyri	R	5.44	0	317	36	–84	–14
Inf. parietal, supramarginal, angular, postcentral, and sup. parietal gyri	R	5.17	0	642	54	–50	54
Triangular and operational inf. frontal and precentral gyri; sup. temporal pole	R	4.98	0.025	129	56	14	2
Precentral and postcentral gyri	R	4.44	0	314	26	–28	56
							
**Positive > negative emotion processing**							
Mid. and sup. occipital gyri; sup. parietal gyrus; precuneus; lingual, fusiform, calcarine, supramarginal, angular, postcentral, and parahippocampal gyri; mid. temporal gyri; cerebellum and vermis	L/R	10.77	0	16 721	–22	–46	–12
Mid. and sup. frontal gyri; precentral gyrus; supplementary motor area; paracentral lobule	L	7.46	0	1120	–20	–10	64
Medial orbital frontal gyrus; medial OFC	L/R	5.90	0.006	173	6	60	–10
Mid. and sup. frontal gyri; precentral gyrus	R	5.80	0	317	26	–10	56
Mid. cingulate gyrus	L	5.53	0.025	129	–14	–28	38
Sup. temporal and supramarginal gyri	L	5.21	0.008	163	–48	–30	22
Sup. parietal, postcentral, and inf. parietal gyri; precuneus	R	5.16	0	333	34	–36	58

*Note.* Hem = hemisphere; OFC = orbitofrontal cortex; inf. = inferior; mid. = middle; sup. = superior; L/R = left/right; *T* = *T*-scores; *k* = cluster size; and *x*, *y*, *z* co-ordinates of peak voxel according to Montreal Neurological Institute (MNI).

Between-group comparisons (two-sample *t*-tests) revealed distinct age-group differences in activation patterns. When processing both positive and negative emotion events, adults showed greater activation in the left hippocampus, precuneus, and temporal regions, whereas children exhibited stronger activation in the right dorsolateral PFC, temporal regions, and bilateral thalamus. For negative > positive emotion events, adults showed greater activation in bilateral lateral PFC and temporal regions, and mid and posterior cingulate cortex, whereas children showed greater activation in bilateral medial PFC and right anterior cingulate cortex (ACC). For positive > negative emotion events, adults showed greater activation in superior medial frontal gyrus and anterior cingulate cortex, whereas children showed greater activation across a more distributed network encompassing the bilateral cingulate cortex, frontal, angular, temporal, and parietal gyri. Peak activations for between-group comparisons are presented in Table 4.

**Table 4:** Peak activation reports for negative, positive, negative > positive, and positive > negative emotion processing, comparing adults > children and children > adults.

	Hem.	*T*	*p*fwe	*k*	MNI		
					*x*	*y*	*z*
**Adults > children**							
** Negative emotion processing**							
Precuneus, hippocampus, and lingual gyrus	L	5.53	0	401	–22	–44	12
Inf. parietal and supramarginal gyrus	L	4.71	0.001	270	–60	–42	48
**Positive emotion processing**							
Precuneus, hippocampus, parahippocampal, and lingual gyri	L	5.22	0	417	–22	–42	12
**Negative > positive emotion processing**							
Mid. and post. cingulate gyri	L/R	5.49	0.02	258	4	–36	32
Angular, inf. and sup. parietal, mid. and sup. occipital gyri	R	4.66	0	407	38	–50	42
Angular, mid. occipital, inf. and sup. parietal gyri	L	4.62	0	806	–40	–46	–16
Inf. and mid. temporal gyri	R	4.59	0.001	266	66	–36	16
Inf. and mid. frontal gyri	L	4.59	0	331	–48	38	14
Inf. and mid. frontal, and sup. frontal gyri	R	4.53	0.011	184	44	50	2
Inf. and mid. temporal gyri	L	4.39	0.009	193	–54	–44	–22
** Positive > negative emotion processing**							
Sup. medial frontal gyri and ACC	L/R	4.86	0.047	131	0	54	22
							
**Children > adults**							
** Negative emotion processing**							
Fusiform, lingual, and inf. occipital gyri	L	5.30	0.002	232	–30	–68	–10
Thalamus and caudate	L/R	4.84	0.001	279	14	–8	18
Fusiform, lingual, cerebellum, and vermis	L/R	4.66	0	292	12	–82	–24
Putamen, pallidum, and caudate	L	4.68	0.018	156	–18	8	6
Precuneus, calcarine, lingual, vermis, and cerebellum	L/R	4.53	0.001	251	–18	–56	6
Mid. and sup. occipital, mid. temporal gyri	R	4.41	0	494	40	–62	8
Operational inf. frontal, mid. frontal, and precentral gyri	R	4.37	0.019	154	34	4	26
Supramarginal and temporal superior gyri	R	4.33	0.017	158	52	–30	16
**Positive emotion processing**							
Inf. and mid. frontal and precentral gyri	R	5.34	0	704	38	6	24
Thalamus and caudate	L/R	5.18	0	310	14	–8	18
Fusiform and lingual gyri; cerebellum	L	4.80	0.049	123	–30	–68	10
Lingual gyrus and cerebellum	R	4.61	0.039	131	12	–82	–24
Mid. and sup. occipital and mid. temporal gyri	R	4.58	0.000	393	42	–78	8
Calcarine and lingual gyri; precuneus; cerebellum	L/R	4.00	0.003	225	–4	–58	8
**Negative > positive emotion processing**							
Medial sup. frontal gyri and anterior cingulate cortex	L/R	4.98	0.027	151	0	54	22
**Positive > negative emotion processing**							
Mid. and post. cingulate	L/R	5.36	0.002	248	4	–36	32
Inf. and sup. parietal, occipital, and angular gyri	L	4.72	0	771	–38	–48	38
Inf., mid. and sup. frontal gyri	L	4.7	0	365	–48	38	14
Angular, inf. and sup. parietal gyri	R	4.67	0	367	38	–48	42
Inf., mid., and sup. frontal gyri	R	4.57	0.011	185	42	50	2
Inf. and mid. temporal gyri	R	4.54	0.002	250	66	–40	–12
Inf. and mid. temporal gyri	L	4.36	0.02	162	–54	–42	–20

*Note.* Hem = hemisphere; ACC = anterior cingulate cortex; inf. = inferior; mid. = middle; sup. = superior; L/R = left/right; T = T-scores; k = cluster size; and x, y, z co-ordinates of peak voxel according to Montreal Neurological Institute (MNI).

### Post hoc: associations between behavioral, cardiac, and neural correlates of emotion regulation and internalizing symptoms

**Figure 4:**
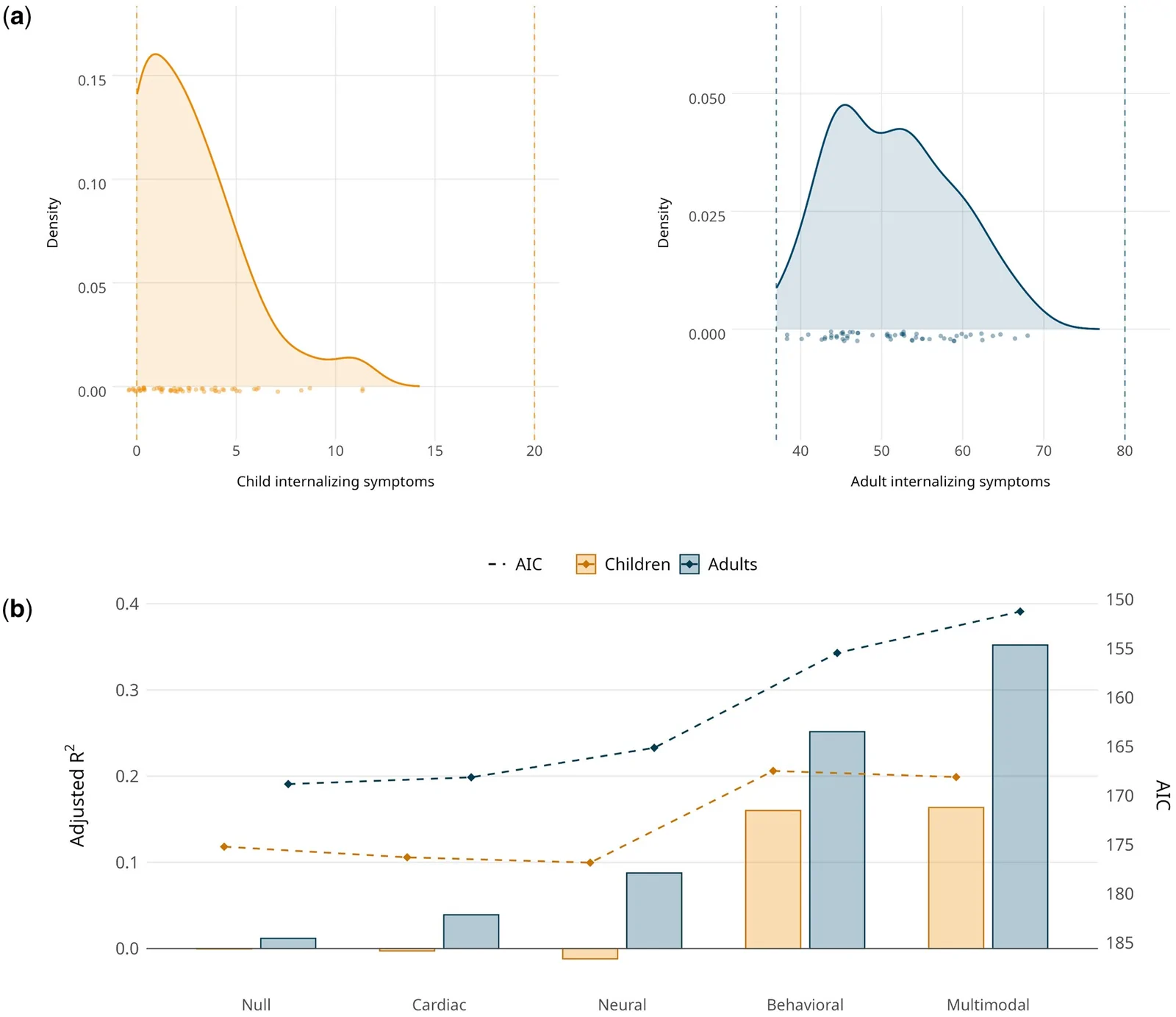
Distribution of internalizing symptoms and model performance across single-modality and multimodal regression models in children and adults. *Note*. (a) Density plots showing the distribution of internalizing symptom scores in children (Strengths and Difficulties Questionnaire, SDQ) and adults (Brief Symptom Inventory, BSI). Dashed vertical lines indicate the minimum and maximum possible scores for each measure. Dots along the *x*-axis represent individual data points. (b) Adjusted *R*^2^ (bars, left *y*-axis) and Akaike Information Criterion (AIC; dashed lines, right *y*-axis) for five regression models predicting internalizing symptoms in children (bars on the left, lower dashed line) and adults (bars on the right, upper dashed line): Null (age and sex only), Cardiac, Neural, Behavioral, and Multimodal. Higher adjusted *R*^2^ indicates greater explained variance; lower AIC indicates better model fit.

Separate regression models were estimated in children and adults to examine associations between behavioral, cardiac, and neural correlates of emotion regulation and internalizing symptoms. The distributions of internalizing symptoms in children and adults are illustrated in Fig. 4a. In children, single-modality models revealed that behavioral correlates were associated with internalizing symptoms, whereas cardiac and neural measures were not. In adults, both behavioral correlates and superior PFC/SMA activity were associated with internalizing symptoms, whereas HRV was not.

In adults, the multimodal model integrating behavioral, cardiac, and neural measures explained more variance in internalizing symptoms and showed improved model fit relative to the single-modality models, as indicated by higher adjusted *R*^2^, lower AIC values, and ANOVA comparisons. In children, the multimodal model explained numerically more variance than the single-modality models; however, improvement relative to the behavioral model was negligible (Δ Adjusted *R*^2^ = 0.003). Lower AIC values and significant ANOVA model comparisons were observed relative to the null, cardiac, and neural models, but not relative to the behavioral model. Table 5 summarizes all model estimates and comparisons; Fig. 4b illustrates adjusted *R*^2^ and AIC across models. Multimodal models without stepwise selection largely replicated the primary findings (see Supplementary Material).

**Table 5:** Linear regression models including behavioral, cardiac, and neural correlates of emotion regulation predicting internalizing symptoms.

	Children					Adults				
Independent variables	Null model	Behavioral model	Cardiac model	Neural model	Multimodal model	Null model	Behavioral model	Cardiac model	Neural model	Multimodal model
Sex (*female as reference group*)										
Male	–0.2932	–0.40897	–0.2793	–0.31319	–0.44810	0.37656	0.41588	0.2747	0.3421	0.31329
Age	0.1031	0.09892	0.1027	0.07683	0.04943	–0.09422	0.02275	–0.1190	–0.1264	–0.04956
Adaptive strategies										
Acceptance		0.34836**			0.36786**					
Positive refocusing		–0.25576*			–0.28072*					
Refocus on planning							–0.27475*			–0.29690*
Positive reappraisal										–0.22070
Putting into perspective										0.23938^†^
Maladaptive strategies										
Self-blame										
Rumination		–0.240501^†^			–0.22072^†^					0.23960^†^
Catastrophizing							0.41115**			0.28904*
Other-blame							0.12598			
HRV			0.1213					–0.2154		–0.27591*
Neural activity clusters during “negative > positive emotion events”										
Left vlPFC				–0.08222	–0.14501					0.14731
Superior PFC/SMA									0.3009*	0.20006
Adjusted *R*^2^	–0.000384	0.1601	–0.002786	–0.01193	0.1636	0.01176	0.2516	0.03914	0.08767	0.3521
**Δ** Adjusted *R*^2^ to complete model	–0.163984	–0.0035	–0.166386	–0.17553		–0.34034	–0.1005	–0.31296	–0.26443	
AIC	175.2181	167.4849	176.3	176.8449	168.1128	168.830	155.4553	168.1973	165.1303	151.2259
**Δ** AIC to complete model	+7.1053	–0.6278	+8.1872	+8.7321		+17.60426	+4.229402	+16.91087	+13.90439	
*F*	0.9887	3.249*	0.9454	0.7681	2.923**	1.339	4.832**	1.774	2.826*	4.098***
Model comparison F to multimodal model	3.7932**	1.2261	4.7126***	4.9167***		4.5821***	2.5739*	4.7265***	4.117**	

*Note*. * *P* <.05. ** *P* <.01. *** *P* <.001. ^†^P <.01

Correlations among predictors were confined within modalities, with no significant associations between behavioral, cardiac, and neural measures. Correlation matrices are reported in the Supplementary Material.

## Discussion

Emotions arise from coordinated behavioral, cardiac, and neural activity, yet their developmental integration remains incompletely understood. Using a movie-watching paradigm, this study demonstrates that affective experiences in children and adults are characterized by coordinated changes in subjective experience, cardiac activity, and engagement of corticolimbic networks. Across age groups, positive and negative emotional content was associated with changes in experienced valence and arousal, heart rate deceleration, and increased activation in prefrontal, temporal, and limbic regions that have previously been implicated in attentional, affective, and regulatory processes. Compared to adults, children showed more positive affect, greater heart rate deceleration, and stronger recruitment of the thalamus and lateral PFC, whereas adults exhibited greater activation in the hippocampus and posterior midline regions. During negative emotion processing specifically, children showed greater medial prefrontal recruitment, whereas adults showed greater lateral prefrontal recruitment. During positive emotion processing specifically, both children and adults engaged ventromedial prefrontal regions. Importantly, combining behavioral, cardiac, and neural measures explained substantially more variance in internalizing symptoms than any single modality alone in the adults, but not in the children’s group. These findings may suggest that associations between state-level cardiac and neural markers and internalizing symptoms differ across age groups.

Children’s and adults’ affect ratings indicate that the movie-watching paradigm effectively elicited emotional responses. Negative emotional content was associated with lower valence and positive emotional content was associated with higher valence in both age groups. Children further reported higher valence at average levels of emotion processing. These findings align with prior evidence for a childhood positivity bias (Walden and Field 1982, Sylvester *et al*. 2016, Vesker *et al*. 2018, Kauschke *et al*. 2019), whereby children tend to evaluate emotional stimuli more positively, including in social and personality judgment (Boseovski 2010). Children and adults also differed in arousal responses: both showed increased arousal to positive content, whereas heightened arousal to negative content emerged only in adults. Children’s stronger arousal response to positive stimuli may preferably enhance attention to positive information (Schimmack and Derryberry 2005, Vogt *et al*. 2008, Vesker *et al*. 2018). These patterns may reflect maturational differences in fronto-limbic circuitry, which continues to develop throughout childhood and adolescence (Casey *et al*. 2011, Silvers *et al*. 2017b) and serve adaptive functions by promoting emotional learning and social engagement early in life (Lagattuta 2014).

At the autonomic level, both positive and negative emotional content was associated with heart rate deceleration in children and adults, consistent with heightened orienting and attentional engagement (Hastings *et al*. 2009, Corcoran *et al*. 2021) and potentially related to regulatory processes discussed in prior literature (Kassam and Mendes 2013). Children exhibited larger heart rate decreases than adults, which may reflect age-group differences in attentional engagement or autonomic responding during emotional processing. This heightened cardiac reactivity converges with children’s greater recruitment of dorsolateral PFC regions, and may be consistent with more effortful or resource-demanding emotion processing mechanisms in childhood (Golkar *et al*. 2012).

Neurally, emotion processing engaged a distributed corticolimbic network in both age groups, including the amygdala, hippocampus, PFC, and TPJ, consistent with prior work on emotion processing, regulation, and social cognition (Saxe 2006, Buhle *et al*. 2014, Alcalá-López *et al*. 2018, Raschle *et al*. 2019). Overlapping activation patterns across groups indicate that children already recruit an adult-like network when processing emotional stimuli (Lobaugh *et al*. 2006, Camacho *et al*. 2019). Nonetheless, age-group differences emerged. Adults showed greater activation in hippocampal, temporal, and posterior midline regions, areas that have previously been linked to memory and self-referential processing (Kennedy and Shapiro 2004, Richardson *et al*. 2004, Jin and Maren 2015, Sweatman *et al*. 2025). In contrast, children exhibited stronger recruitment of dorsolateral prefrontal, temporal, and thalamic regions, which have been linked to sensory integration and regulatory control (Golkar *et al*. 2012, Sherman 2016). Together, these findings reveal age-group differences in the regions recruited during emotion processing, with children showing relatively greater engagement of regions previously associated with sensory integration and regulatory control and adults of regions related to memory and self-referential processes.

Negative emotion processing was characterized by increased activation in prefrontal and temporal regions relative to positive emotion processing in both groups. Our finding of enhanced lateral PFC activation, a region implicated in cognitive control and emotion regulation (Rubia 2013), is broadly consistent with prior work showing increased prefrontal recruitment during negative emotional processing (Ochsner and Gross 2008, Ochsner *et al*. 2012, Buhle *et al*. 2014). Because participants were not instructed to engage in specific regulation strategies during viewing, the extent to which these activation patterns reflect emotion regulation processes cannot be directly determined. Moreover, increased activation in temporal regions, including the TPJ, further suggests heightened demands on mentalizing and perspective taking processes during negative emotional content (Saxe 2006, Borbás *et al*. 2021). Although children and adults showed partially overlapping activation patterns, adults recruited lateral PFC and cingulate regions more strongly, whereas children showed greater engagement of medial prefrontal and cingulate regions. These age-group differences in medial and lateral prefrontal recruitment broadly align with prior developmental work demonstrating an increasing reliance on deliberate cognitive control during emotion regulation across development (McRae *et al*. 2012, Silvers *et al*. 2017a).

Positive emotion processing was associated with greater activation in the ventromedial PFC (vmPFC) relative to negative emotion processing in both groups. Experiencing more positive valence and increased vmPFC activity during positive emotion processing, a region implicated in safety signaling and positive affect tracking (Kong *et al*. 2014, Harrison *et al*. 2017), suggests that positive emotional content transmits safety signals that evoke positive affective changes (Harrison *et al*. 2017). While both age groups engaged a partially overlapping set of regions, children showed greater engagement of a distributed network, including bilateral cingulate, lateral frontal, parietal, and temporal regions, consistent with stronger engagement with positive emotional content earlier in development (Boseovski 2010, Vesker *et al*. 2018) and in line with children’s stronger arousal response to positive emotional content discussed earlier.

The associations between multimodal correlates and internalizing symptoms differed across groups. In adults, integrating behavioral, cardiac, and neural indices explained more variance in internalizing symptoms and showed better fit relative to any single modality models, and these predictors were not correlated, consistent with the possibility that behavioral, cardiac, and neural measures may capture partially distinct aspects of socioemotional functioning and mental health (Waugh *et al*. 2012, Dich *et al*. 2019). In children, however, the multimodal model substantially outperformed the null, cardiac, and neural models, but not the behavioral model, indicating that the specific cardiac and neural indices used here did not add incremental explanatory value for internalizing symptoms beyond questionnaire-based emotion regulation strategies in this community sample. Several factors may account for this pattern. First, the low and right-skewed symptom distribution in the child sample may have limited statistical power to detect cardiac and neural contributions. Second, the cardiac and neural predictors used here may not have captured individual differences most relevant to trait-level internalizing symptoms in childhood. Third, associations between task-evoked cardiac and neural responses and trait-level symptoms may differ across age groups, potentially reflecting ongoing maturation of autonomic and neural systems during childhood (Casey *et al*. 2019, Harteveld *et al*. 2021). Consistent with this possibility, task-evoked fMRI activity in children has been shown to exhibit lower reliability than in adults (Elliott *et al*. 2020, Kennedy *et al*. 2022), which may reduce its utility for predicting trait-level outcomes. Together, these findings highlight the need for future work using alternative cardiac and neural measures and spanning a broader symptom range, and suggest that associations between state-level physiological responses and trait-level symptoms may vary across age groups.

Beyond model performance, the specific correlates associated with internalizing symptoms differed by age group. In adults, greater use of *refocus on planning* and greater HRV were associated with lower internalizing symptoms, in line with previous literature (Aldao *et al*. 2010, Mulcahy *et al*. 2019, Niu *et al*. 2023). In children, greater *acceptance* was associated with higher internalizing symptoms, suggesting that it reflects passive rather than active regulatory engagement (Garnefski and Kraaij 2006), whereas greater *positive refocusing* was associated with lower symptoms. These findings suggest that the associations between multimodal correlates and mental well-being change across age groups.

The present findings extend a growing body of work employing naturalistic movie-watching paradigms to investigate socioemotional processes across behavioral, cardiac, and neural levels (Mauss *et al*. 2005, Nummenmaa *et al*. 2012, Balconi *et al*. 2014, Kirk and Robinson 2024). By analyzing all three modalities within the same paradigm and across two distinct developmental periods, this study demonstrates that such paradigms can reliably evoke coordinated responses across all levels of analysis and in samples spanning childhood and adulthood. One inherent constraint of naturalistic designs is that participants were not instructed to engage specific emotion regulation strategies during viewing, such that regulatory engagement remains implicit and cannot be directly verified. Consequently, prefrontal recruitment and heart rate deceleration cannot be directly interpreted as evidence of emotion regulation, underscoring the value of combining naturalistic and task-based paradigms in future work to disentangle implicit from explicit and instructed regulation.

Further limitations should be acknowledged. First, emotional events were defined based on the main character’s expressions, and emotional cues from other characters that may have influenced participants’ responses were not modeled in the analyses. In addition to that, the movie on which the paradigm is based on features a child protagonist and is targeted toward younger audiences, which may have strengthened children’s engagement with the movie and identification with the protagonist, and contributed to some of the observed group differences. Second, the two-group design, with no data collected during adolescence, did not allow for continuous modeling of age-related trajectories across development. Third, data were collected from community rather than clinical samples, with particularly low symptom scores in children, limiting the generalizability of findings to clinical disorders. This limitation was further complicated by the use of different symptom measures across age groups (adult symptoms were derived from self-reported, sex-, and age-adjusted t-scores, whereas child symptoms reflected parent-reported scores), which constrains the comparability of symptom associations across groups. Thus, cross-group comparisons should be interpreted cautiously. Last, behavioral affect ratings and multimodal measures were collected in independent samples, preventing within-subject integration across all levels. Future studies that combine behavioral, autonomic, and neural measures within the same individuals, sampling across childhood, adolescence, and adulthood, and using developmentally consistent assessment tools in clinically diverse samples are needed.

## Conclusion

In sum, the present findings demonstrate that socioemotional experiences are associated with coordinated behavioral, cardiac, and neural responses across childhood and adulthood. Combining these indices accounted for additional variance in the explanation of internalizing symptoms in adults relative to single-modality models, whereas cardiac and neural measures contributed comparatively little beyond behavioral measures in children. These findings highlight the value of multimodal approaches and naturalistic paradigms for studying socioemotional functioning and its associations with mental well-being across development.

## Supplementary Material

nsag050_Supplementary_Data

## Data Availability

The behavioral and cardiac data underlying this article are available on OSF (https://osf.io/mdtjp/). Unthresholded T-maps of fMRI analyses conducted in this article are available on NeuroVault (https://neurovault.org/collections/WRIGYWKR/).
