# Supplementary material for: Multimodal correlates of socioemotional movie-watching and their associations with internalizing symptoms in childhood and adulthood
Source: Soc Cogn Affect Neurosci. 2026 Jun 23;21(1):nsag050. doi: 10.1093/scan/nsag050 (PMC13427769; doi:10.1093/scan/nsag050)
Supplement: nsag050_Supplementary_Data [file nsag050_supplementary_data.zip › SM_updated_proofs.docx]

**SUPPLEMENTARY MATERIALS**

**Multimodal correlates of socioemotional movie-watching and their associations with internalizing symptoms in childhood and adulthood**

Sofia Scatolin, Elena Federici, Plamina Dimanova, Réka Borbás,

Mirjam Habegger, & Nora Maria Raschle

**Participant recruitment and data collection procedure – Study Part 1:**

**Child group:** Teachers and schools were asked about their interest in participating in the study. After signing up to participate, schools agreed to the standard procedures, which included ensuring that all families receive information on the study aims and content. Answer choices, age, and sex, but no identifiable personal data from the children were collected. Families that did not want their children’s answers to be included in the group analysis were given the option to opt out. Participants received small gifts after data collection.

**Adult group:** Participants were recruited via online university mailing lists and consented to participate in the study, prior to answering any questions. Data was collected using RedCap (https://project-redcap.org/). Answer choices, age, and sex, but no identifiable personal data were collected. Participants were reimbursed with university research points or a monetary amount of CHF 20.

**Participants’ education and country of origin – Study Part 2**

Highest education level was assessed using the International Standard Classification of Education (ISCED; UNESCO, 2012). The average parental ISCED level for children was 5.9 (SD = 1.9, range = 2–8), corresponding to tertiary to bachelor’s level education, while the adult sample ISCED level averaged 4.2 (SD = 1.6, range = 3–7), corresponding to post-secondary to non-tertiary education.

Although all participants resided in Switzerland, countries of origin varied. Among the children’s families, 27 were from Switzerland, 22 from Switzerland and other European countries, 16 from Switzerland and non-European countries, 26 from other European countries, 4 from European and non-European countries, 3 from non-European countries, and 12 did not report. For the adults, 40 were of Swiss origin, 5 from Switzerland and other European countries, 3 from Switzerland and non-European countries, 13 from other European countries, 1 from non-European countries, and 2 did not report.

**Movie-watching paradigm evaluation**

**Content analysis**

The content of movie-watching paradigm was analyzed using EmoCodes, an externally validated frame-by-frame system designed for coding the affective content of complex stimuli (Camacho et al., 2022). Three independent raters coded the emotions and affect intensity displayed by eight characters. Emotions coded included anger, sadness, fear, happiness, surprise, disgust, and shame. The codes were processed using the *emocodes* Python library (version: 1.0; Camacho et al., 2022). Errors when coding, defined as clear missing ratings for characters or emotions for prolonged periods of time and indicated by low consensus (<80%) among raters, occurred in 0.027% (5 out of 187) of coded variables. These errors were corrected based on the codes of other raters. Post-correction, there was an average inter-rater consensus of 98.46% (SD: 3.28).

The main character’s emotions were further categorized into positive (codes for happiness and surprise) and negative (codes for anger, sadness, fear, disgust, and shame) emotions. Specifically, the onsets and durations of events in which the main character displayed positive or negative emotions, based on unanimous agreement among raters, were extracted. This process allowed the establishment of two regressors: 'Positive Emotions' and 'Negative Emotions’. Any segments where these conditions intersected were identified, with the intention to exclude them, although no such segments were found. Furthermore, segments that simultaneously presented conflicting affects between the main character and any other character (e.g., such as a segment towards the conclusion, when the main character's negative emotions coincided with her mother's positive emotions, lasting 5 seconds) were excluded to avoid ambiguity in emotional valence assignment.

**Questionnaire:**


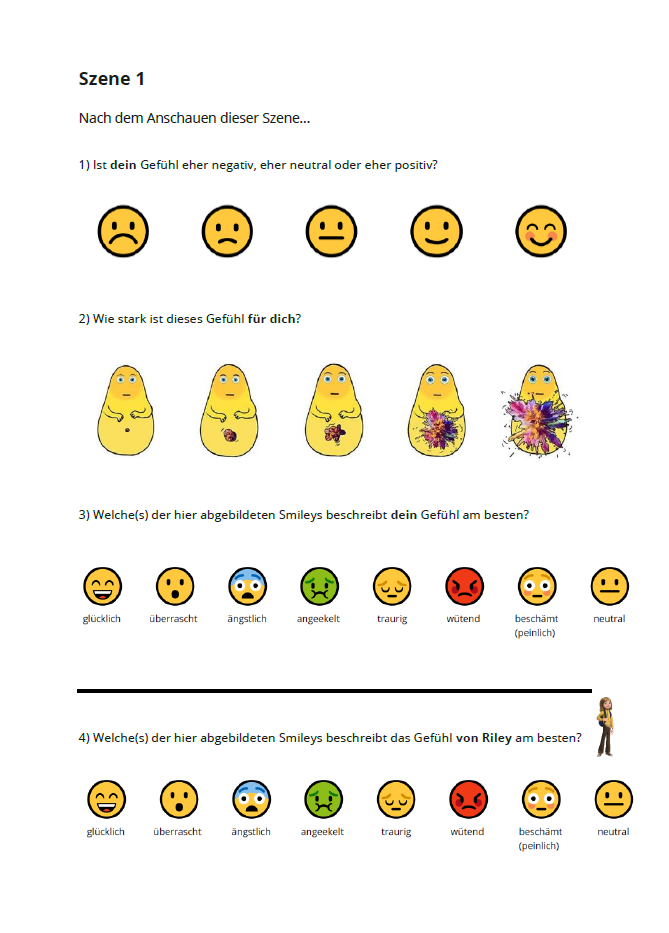


**Supplemental Figure 1*.*** Age-appropriate questionnaire applied to children and adults. The questionnaire was administered after each of the 17 movie scenes and included four items: (1) "Is **your** feeling rather negative, rather neutral, or rather positive?" assessed on a 5-point scale ranging from negative to positive, depicted using facial emoji expressions; (2) "How strong is this feeling **for you**?" assessed on a 5-point scale ranging from low to high intensity, depicted using the character progressing from calm to high arousal; (3) "Which of the smileys shown here best describes **your** feeling?" with eight response options (happy, surprised, anxious, disgusted, sad, angry, ashamed/embarrassed, neutral); and (4) "Which of the smileys shown here best describes **Riley's** feeling?" with the same eight response options. For the adult group, the questionnaire was adapted to an online environment while retaining identical item content and response scales.

**Neuroimaging and Cardiac Data Acquisition**

In Study Part 2, participants underwent functional magnetic resonance imaging (fMRI), on a 3T General Electric Signa MR scanner equipped with a 48-channel head coil, alongside simultaneous photoplethysmography (PPG) recorded at 100 Hz via a pulse oximeter to assess cardiac responses while they watched the movie-watching paradigm. Functional images were acquired using an echo-planar imaging (EPI) pulse sequence sensitive to T2* contrast, with the following parameters: field of view = 240mm, TR = 2203.0ms, TE = 20.3ms, 41 slices, slice thickness = 2mm, voxel size = 2.5 x 2.5 x 2.0mm, and 342 volumes. Dummy scans were included to account for equilibration effects and were excluded from further analyses. Before scanning, participants received age-appropriate instructions and familiarization training to minimize head motion. The paradigm was presented on an MR-compatible LCD screen, viewed via a mirror mounted on the MR head coil. Audio was delivered through noise-attenuating, MR-compatible headphones.

**Results of Study Part 2 – Neural correlates**

**Conjunction Analyses between Child and Adult Groups**

**Negative emotion processing**

Bilateral amygdala, hippocampus, precuneus, cuneus, thalamus, temporal pole, cerebellum, and precentral, frontal inf. (triangular and orbital), parahippocampal, calcarine, lingual, occipital (inf., mid., and sup.), fusiform, postcentral, supramarginal, angular, Heschl’s, and temporal (inf, mid., and sup.) gyri.

Right Rolandic operculum, supplementary motor area, and paracentral lobule.

Left posterior and lateral OFC.

**Positive emotion processing**

Bilateral amygdala, hippocampus, precuneus, cuneus, thalamus, temporal pole, paracentral lobule, cerebellum, and precentral, frontal inf. (triangular and orbital), parahippocampal, calcarine, lingual, occipital (inf., mid., and sup.), fusiform, postcentral, sup. parietal, supramarginal, angular, Heschl’s, and temporal (inf, mid., and sup.) gyri.

Right: Rolandic operculum.

Left: frontal mid., frontal sup., and frontal sup. (medial) gyri.

**Negative > positive emotion processing**

Bilateral supplementary motor area, temporal pole, and frontal inf. (opercular), mid. cingulate, inf. parietal, supramarginal, and sup. temporal gyri.

Right: Rolandic operculum.

Left frontal insula, inf. (triangular and orbital), sup. frontal, sup. frontal (medial), and angular gyri.

**
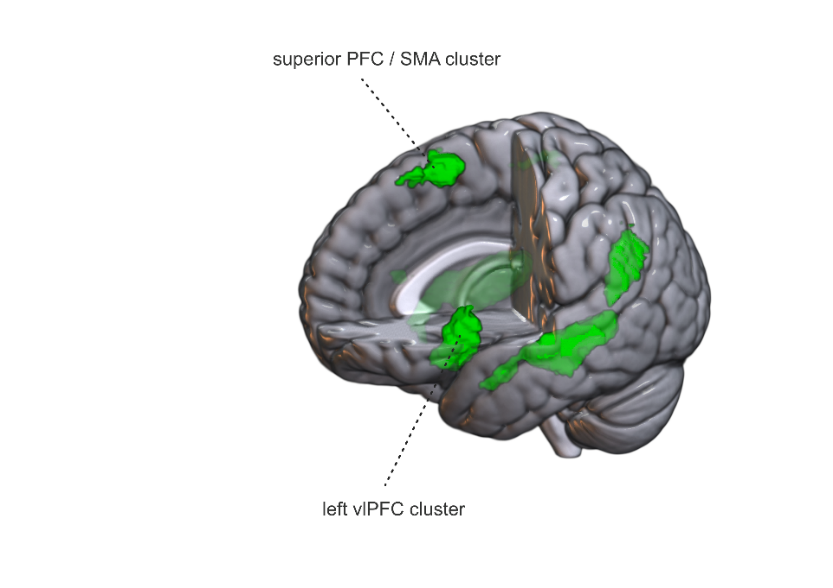
**

**Supplemental Figure 2.** Voxels commonly activated by children and adults during ‘negative > positive emotion processing’. The superior PFC/SMA and left vlPFC clusters were used in post-hoc analyses.

**Positive > negative emotion processing**

Bilateral precentral, frontal (mid. and sup.), medial orbital frontal, parahippocampal, calcarine, cuneus, lingual, occipital (inf., mid. and sup.), fusiform, postcentral, superior parietal, inferior parietal, angular, precuneus, inferior temporal (inf. and mid.) gyri, paracentral lobule, mid-cingulate, cerebellum, and vermis.

Left: supplementary motor area, supramarginal, hippocampus.

**Post-hoc: Associations between Correlates of Emotion Regulation and Internalizing Symptoms without Stepwise Selection**

**Supplemental Table 3.** Linear regression models including all behavioral, cardiac, and neural correlates of emotion regulation predicting internalizing symptoms. These analyses were conducted to assess robustness of the main findings and were exploratory in nature.

|  | **Children** |  | | |  |  | |  | | **Adults** | | | | | | |
| --- | --- | --- | --- | --- | --- | --- | --- | --- | --- | --- | --- | --- | --- | --- | --- | --- |
| **Independent variables** | Null model | | Behavioral model | Cardiac  model | | | Neural  model | | Multimodal model | | Null model | Behavioral  model | Cardiac model | Neural  model | Multimodal  model |  |
| Sex (female as reference group) |  | |  |  | | |  | |  | |  |  |  |  |  |  |
| Male | -0.2932 | | -0.40325 | -0.2793 | | | -0.30957 | | -0.43620 | | 0.37656 | 0.57469 | 0.2747 | 0.33992 | 0.331254 |  |
| Age | 0.1031 | | 0.13220 | 0.1027 | | | 0.07252 | | 0.05499 | | -0.09422 | 0.04120 | -0.1190 | -0.12095 | -0.046414 |  |
| Adaptive strategies |  | |  |  | | |  | |  | |  |  |  |  |  |  |
| Acceptance |  | | 0.36832* |  | | |  | | 0.38495* | |  | -0.01208 |  |  | -0.004838 |  |
| Positive refocusing |  | | -0.26096 . |  | | |  | | -0.27403 . | |  | -0.05347 |  |  | -0.148087 |  |
| Refocus on planning |  | | -0.07374 |  | | |  | | -0.08380 | |  | -0.30218 * |  |  | -0.287579 * |  |
| Positive reappraisal |  | | -0.06744 |  | | |  | | -0.10811 | |  | -0.12473 |  |  | -0.196524 |  |
| Putting into perspective |  | | 0.06413 |  | | |  | | 0.10874 | |  | 0.21053 |  |  | 0.323476 * |  |
| Maladaptive strategies |  | |  |  | | |  | |  | |  |  |  |  |  |  |
| Self-blame |  | | -0.05676 |  | | |  | | -0.10485 | |  | -0.16290 |  |  | -0.102873 |  |
| Rumination |  | | -0.23839 . |  | | |  | | -0.23783 . | |  | 0.11171 |  |  | 0.196503 |  |
| Catastrophizing |  | | 0.14496 |  | | |  | | 0.19541 | |  | 0.43480 ** |  |  | 0.347300 |  |
| Other-blame |  | | -0.16183 |  | | |  | | -0.15803 | |  | 0.14736 |  |  | 0.070135 |  |
| HRV |  | |  | 0.1213 | | |  | | 0.10174 | |  |  | -0.2154 |  | -0.216269 |  |
| Neural activity clusters during ‘negative > positive emotion processing’ |  | |  |  | | |  | |  | |  |  |  |  |  |  |
| Left vlPFC |  | |  |  | | | -0.07363 | | -0.14786 | |  |  |  |  | 0.144003 |  |
| Superior PFC/SMA |  | |  |  | | | -0.01781 | | -0.08213 | |  |  |  |  | 0.243417 . |  |
| Adjusted R2 | -0.000384 | | 0.09666 | -0.002786 | | | -0.03012 | | 0.0842 | | 0.01176 | 0.215 | 0.03914 | 0.07508 | 0.3175 |  |
| Δ Adjusted R2 to multimodal model | -0.084584 | | +0.01246 | -0.086986 | | | -0.11432 | |  | | -0.30574 | - 0.1025 | -0.27836 | - 0.24242 |  |  |
| AIC | 175.2181 | | 176.7847 | 176.3 | | | 178.8323 | | 179.7343 | | 168.830 | 163.1138 | 168.1973 | 166.841 | 157.0816 |  |
| Δ AIC to complete model | -4.5162 | | -2.9496 | -3.4343 | | | -0.902 | |  | | +11.74861 | +6.032212 | +11.05521 | +9.75954 |  |  |
| F | 0.9887 | | 1.574 | 0.9454 | | | 0.5688 | | 1.387 | | 1.339 | 2.419 * | 1.774 | 2.157 . | 2.894 ** |  |
| Model comparison F to multimodal model | 1.4387 | | 0.7823 | 1.4835 | | | 1.6865 | |  | | 3.0536** | 3.3044* | 3.0027** | 2.883** |  |  |

Note. . *p* < .1; * *p* < .05; ** *p* < .01; *** *p* < .001

**Supplemental Table 3.** Pearson correlation matrices of behavioral, cardiac, and neural predictors entered in the multimodal regression models, separately for children and adults.

| ***Children*** | | | | | | | | | | | | |
| --- | --- | --- | --- | --- | --- | --- | --- | --- | --- | --- | --- | --- |
| Variable | 1 | 2 | 3 | 4 | 5 | 6 | 7 | 8 | 9 | 10 | 11 | 12 |
| 1. Acceptance | — |  |  |  |  |  |  |  |  |  |  |  |
| 2. Positive refocusing | 0.15 | — |  |  |  |  |  |  |  |  |  |  |
| 3. Refocus on planning | 0.20 | 0.01 | — |  |  |  |  |  |  |  |  |  |
| 4. Positive reappraisal | 0.09 | 0.17 | 0.30* | — |  |  |  |  |  |  |  |  |
| 5. Putting into perspective | 0.41** | 0.34** | 0.19 | 0.29* | — |  |  |  |  |  |  |  |
| 6. Self-blame | 0.33** | -0.14 | 0.23 | 0.11 | 0.28* | — |  |  |  |  |  |  |
| 7. Rumination | 0.19 | 0.13 | 0.01 | 0.24 | 0.22 | -0.09 | — |  |  |  |  |  |
| 8. Catastrophizing | 0.14 | -0.21 | 0.02 | 0.26* | -0.16 | 0.13 | 0.19 | — |  |  |  |  |
| 9. Other-blame | 0.18 | -0.09 | -0.22 | -0.05 | 0.01 | -0.21 | 0.24 | 0.16 | — |  |  |  |
| 10. HRV (RMSSD) | -0.01 | -0.15 | 0.05 | 0.13 | 0.11 | 0.18 | 0.00 | 0.10 | -0.04 | — |  |  |
| 11. vlPFC (neg > pos) | 0.08 | -0.15 | -0.16 | 0.04 | 0.12 | 0.05 | 0.11 | 0.22 | 0.11 | 0.07 | — |  |
| 12. Superior PFC/SMA (neg > pos) | -0.04 | -0.02 | -0.28* | -0.19 | -0.06 | -0.30* | -0.07 | 0.14 | 0.10 | 0.12 | 0.55*** | — |
|  |  |  |  |  | ***Adults*** |  |  |  |  |  |  |  |
| 1. Acceptance | — |  |  |  |  |  |  |  |  |  |  |  |
| 2. Positive refocusing | 0.07 | — |  |  |  |  |  |  |  |  |  |  |
| 3. Refocus on planning | 0.33* | 0.10 | — |  |  |  |  |  |  |  |  |  |
| 4. Positive reappraisal | 0.46*** | 0.14 | 0.45*** | — |  |  |  |  |  |  |  |  |
| 5. Putting into perspective | 0.41** | 0.38** | 0.35** | 0.36** | — |  |  |  |  |  |  |  |
| 6. Self-blame | 0.22 | -0.13 | 0.08 | 0.04 | 0.12 | — |  |  |  |  |  |  |
| 7. Rumination | 0.09 | -0.09 | 0.17 | 0.23 | -0.11 | -0.00 | — |  |  |  |  |  |
| 8. Catastrophizing | -0.17 | -0.12 | -0.10 | -0.14 | -0.32* | 0.23 | 0.42*** | — |  |  |  |  |
| 9. Other-blame | -0.21 | 0.03 | -0.11 | -0.06 | -0.20 | -0.05 | 0.11 | -0.06 | — |  |  |  |
| 10. HRV (RMSSD) | 0.05 | 0.31* | -0.05 | -0.11 | 0.15 | 0.13 | 0.03 | -0.06 | 0.01 | — |  |  |
| 11. vlPFC (neg > pos) | -0.14 | 0.21 | -0.09 | -0.04 | -0.13 | 0.06 | -0.16 | 0.08 | 0.22 | 0.09 | — |  |
| 12. Superior PFC/SMA (neg > pos) | -0.09 | 0.24 | -0.08 | -0.07 | -0.18 | 0.05 | -0.05 | 0.10 | 0.13 | -0.03 | 0.48*** | — |

Note. *p* < .1; * *p* < .05; ** *p* < .01; *** *p* < .001
